# State-of-the-art predictive modeling of heavy metal ions removal from the water environment using nanotubes

**DOI:** 10.1038/s41598-023-38442-w

**Published:** 2023-07-14

**Authors:** Zeinab Ghasemi, Farzaneh Farzad, Ameneh Zaboli, Ali Zeraatkar Moghaddam

**Affiliations:** Department of Chemistry, University of Birjand, Birjand, Iran

**Keywords:** Cheminformatics, Environmental chemistry, Physical chemistry

## Abstract

In this research, molecular dynamics (MD) simulation is used to investigate the efficiency of carbon nanotubes (CNT) and boron nitride nanotubes (BNNT) in removing lead ions from contaminated waters. Then the effect of functionalizing nanotubes with –COO– and COOH– functional groups and the nanotubes’ absorption performance of two different concentrations of lead ions are studied. To better evaluate adsorption process, the set of descriptors, such as interaction energies, radial distribution function, etc., are calculated. The MD results show that the absorption performance is significantly improved by modifying the surface of CNT and BNNT with functional groups. In addition, the adsorption capacity increases in higher concentrations of Pb ions at BNNTCOO– and CNTCOOH systems. The interaction energy of BNNTCOO– with a concentration of 50 lead ions is − 2879.28 kJ/mol, which is about 106 kJ/mol more negative than BNNTCOO– at a concentration of 20 lead ions. Also, it is observed that the functionalization of both nanotubes with –COO– increases their absorption capacity. The obtained results from this study provide significant information about the mechanisms of lead adsorption on the surface of nanotubes.

## Introduction

In the recent decade, industrial and technological development have caused the quality and comfort of human life to become better^1^. Despite the many advantages of industrial products, creating these products causes the entry of heavy metals such as iron, zinc, and lead into the air, water, and soil^2^. Clean water is essential for the life of all creatures, and too much release of such heavy metals into water will have dangerous effects on human health^3^. Therefore, using novel techniques to eliminate heavy metals from the environment is necessary^4–7^.

Lead (Pb) ion is a hazardous and non-biodegradable heavy metal that can be accumulated in the body. Excessive amounts of Pb ions in drinking water can cause cancer, damage to the nervous system, anemia, kidney disease, and retardation. Thus, the removal of Pb ions is necessary for environmental protection. So far, many methods have been used to eliminate lead, such as ion exchange, electrochemical removal, chemical deposition, adsorption, and so on^8^.

The adsorption method, with the high removal capability of contaminants even at very low concentrations and the availability of raw materials to make adsorbents, is considered a suitable technique for removing heavy metals from water^9–11^. Today, nanomaterials have received much attention as adsorbents^12–14^ owing to their unique properties, such as large surface area, small size, high surface-to-volume ratio, good catalytic potential, and high reactivity^9,10,15^.

Various nanomaterials like dendrimers, zeolites, and carbon nanotubes (CNTs) have been presented to remove heavy metals from water^16^. CNT exists in two forms: single-walled CNT(SWCNT) and multi-walled CNT(MWCNT)^17^. Researchers have used both types of CNT as adsorbents, and their results have shown that SWCNT has a higher adsorption capacity than MWCNT. CNT surfaces are highly hydrophobic, which with functionalization can overcome this problem^13,14^.

Functional groups such as carboxylic (–COO–), hydroxyl (–OH), and amide (–NH2) on the CNT surface increase the adsorption capacity of the nanotube.

Anitha and coworkers^18^compared the adsorption capacity of CNT and functionalized CNT with carboxylic(–COO–), hydroxyl(–OH), amide (–NH2) groups to remove cadmium (Cd2+), lead (Pb2+), copper (Cu2+). They showed the CNT surface in the presence of the carboxylic functional group has a higher adsorption capacity than the hydroxyl and amide functional groups and pristine CNT. Also, Sadegh et al.^19^ examined the ability of CNTs to remove of several contaminants, methods of functionalizing CNTs surfaces, and the effect of functional groups on increasing adsorption capacity.

BNNTs are an alternative to CNTs due to their high torsion resistance, conductivity, and thermal stability at high temperatures^20–22^.

Functionalized BNNT, similar to functionalized CNT, has a higher adsorption capacity than its pristine type^23^. Azamat et al.^24^ investigated the ability of CNT and BNNT as membranes to remove Pb ions, Cu, and Hg ions in the presence of an electric field and found that BNNT had a higher adsorption capacity than CNT due to hydrogen bonding with water molecules. Aroche et al.^23^ showed that the external electric field affects the performance of CNT and BNNT in the removal of Zn, Cd, Hg, Pb, and Fe ions.

Here, the effect of functionalization carbon and boron nitride nanotubes with COO^-^ and COOH functional groups in the removal of lead with different concentrations in the absorption process was investigated. Our results showed that with increasing Pb ions concentration, the adsorption capacity increases. Also, functional groups had a significant effect on increasing the absorption capacity.

## Methods

**Figure 1:**
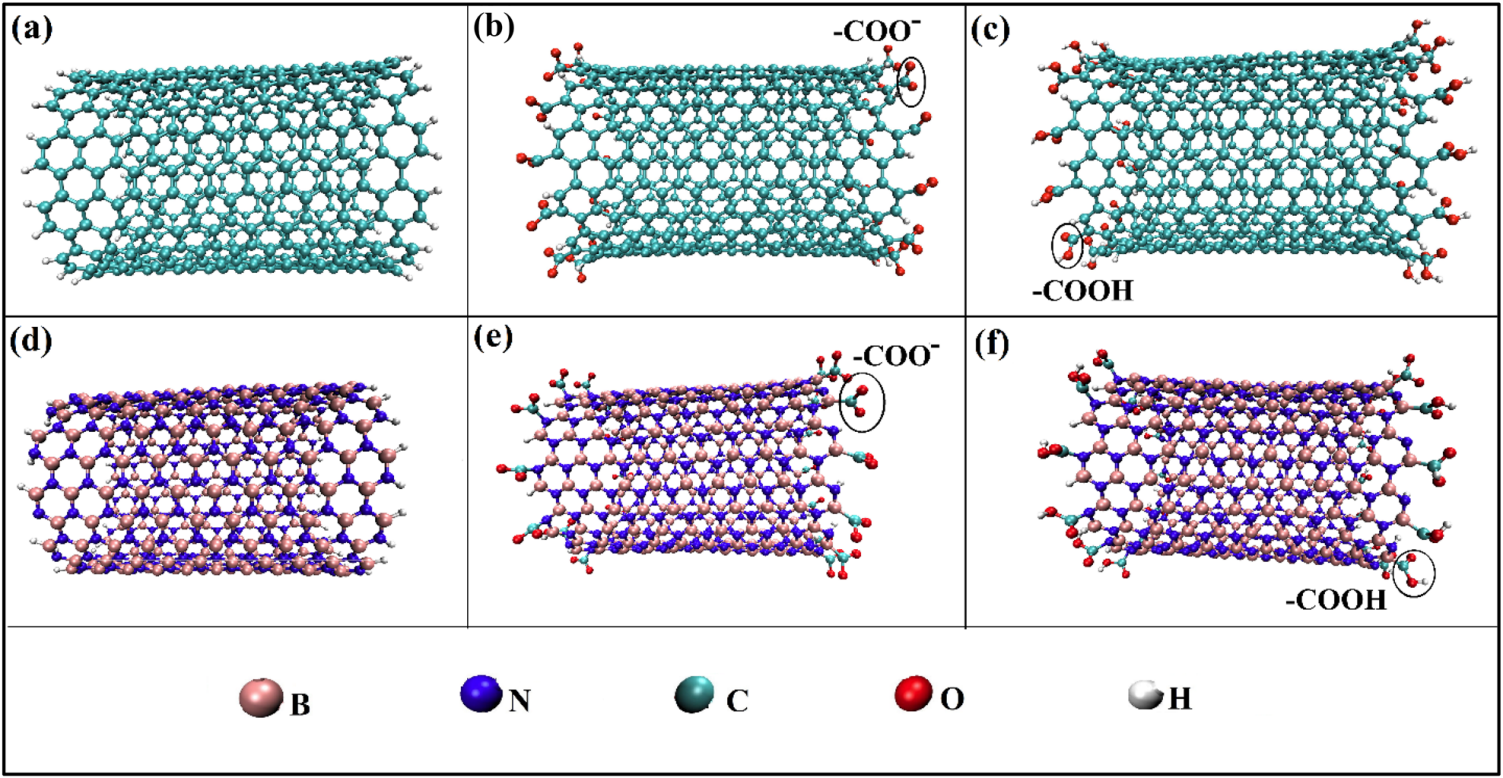
The structure of (**a**) CNT, (**b**) CNTCOO^−^, (**c**) CNTCOOH, (d) BNNT, (**e**) BNNTCOO^−^, (**f**) BNNTCOOH.

The adsorption of different lead concentrations on the CNT, BNNT, functionalized CNT (FCNT), and BNNT (FBNNT) was investigated using molecular dynamics (MD) simulation. The VMD software was utilized to model single-walled armchair structures of CNT and BNNT, both possessing a (12,12) chirality, a length of approximately 2.5 nm, and a diameter of roughly 1.64 nm. CNT contains 504 carbon atoms and 48 hydrogen atoms, and BNNT contains 240 boron atoms, 240 nitrogen atoms, and 48 hydrogen atoms. The twelve of –COO– and –COOH were placed at both ends of the nanotube for functionalization, as shown in Fig. 1.

**Figure 2:**
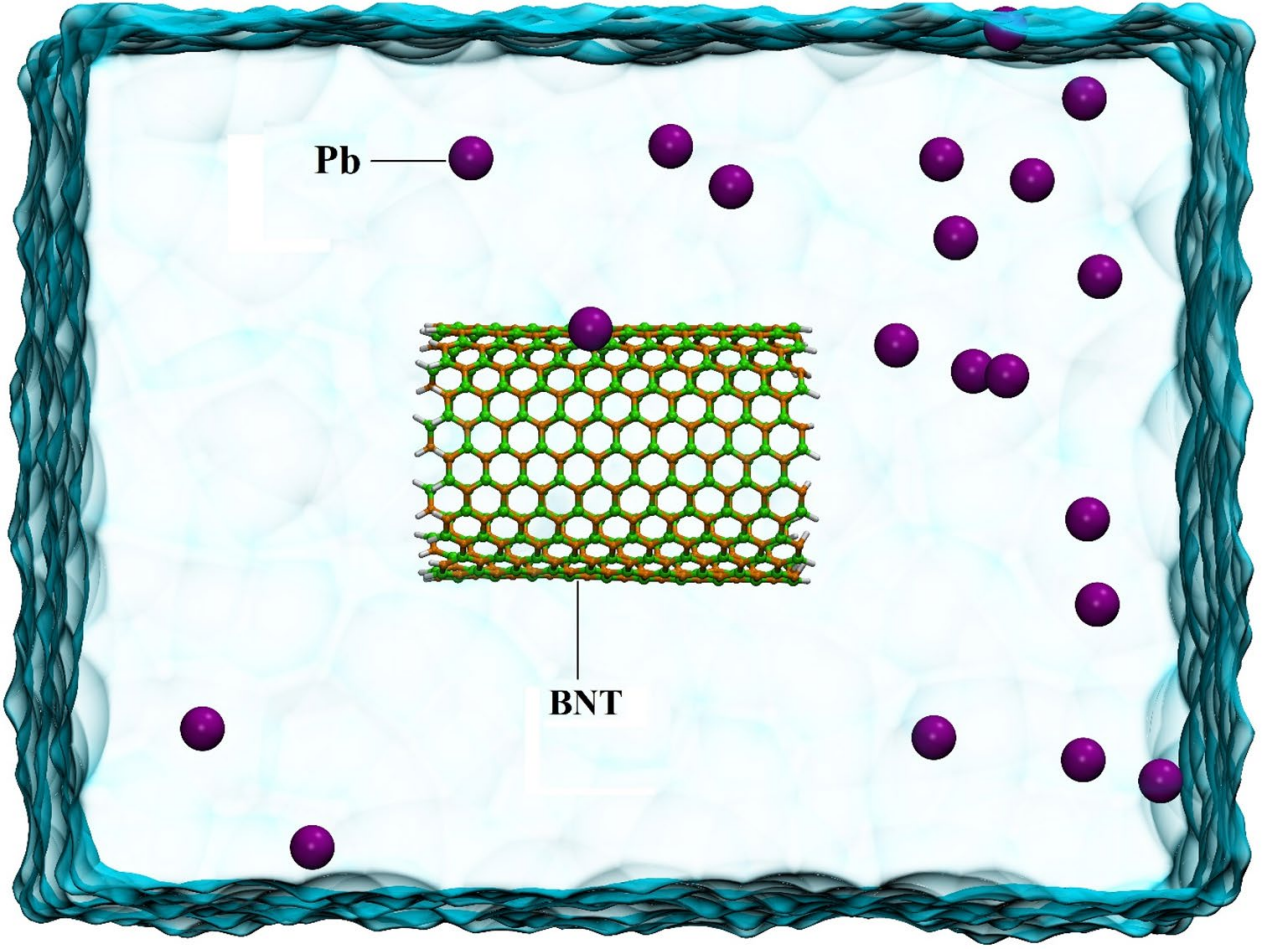
Initial snapshot of MD simulation box for BNNT containing 20 Pb ions system.

Six boxes with dimensions of 6 × 6 × 8 nm^3^ were designed, in which CNT, CNTCOO–, CNTCOOH, BNNT, BNNTCOO–, and BNNTCOOH nanotubes were placed separately in their center, and then 20 Pb ions were randomly added to each system at an average distance of 7 to 30 Å. In order to investigate the performance of adsorbents in adsorbing ions at higher concentrations, six other systems were designed, and 50 lead ions are randomly located in them. In the following, water molecules were explicitly included in the systems using the TIP3P model^25^. Force field parameters for boron nitride nanotube^26^and lead ions^27^ are extracted from previous studies, and for the other components of the simulation system are taken from the CHARMM27 force field^28^. Then, the steepest descent algorithm is executed to minimize the energy of the simulation systems^29^. MD simulation for all systems is performed at canonical and isobaric-isothermal conditions at 310 K and 1 bar by utilizing the GROMACS package Version 5.1.4^30^. Temperature is controlled by using the V-rescale thermostat and pressure using the Berendsen barostat to integrate Newton’s movement equations using the leap-frog algorithm in the time step of 2 fs^24,25^. In order to keep all links in their equilibrium positions, the LINCS algorithm is employed and periodic border conditions apply in all three orientations. Electrostatic interactions are calculated with the particle mesh Ewald (PME) method, and MD simulations for all systems run for 75 ns. The initial state of one of the studied systems has shown in Fig. 2, and the details of the simulation systems have given in Table 1.

**Table 1:** Detail of the simulation boxes which used in this study.

System	Nanotube	Functional group	Number of Pb ions	Number of water molecules
CNT@20Pb	CNT	–	20	9120
CNTCOO^-^@20Pb	CNT	–COO^-^	20	9101
CNTCOOH@20Pb	CNT	–COOH	20	9099
BNNT@20Pb	BNNT	–	20	9117
BNNTCOO^-^@20Pb	BNNT	–COO^−^	20	9105
BNNTCOOH@20Pb	BNNT	–COOH	20	9089
CNT@50Pb	CNT	–	50	9009
CNTCOO^-^@50Pb	CNT	–COO^−^	50	9024
CNTCOOH@50Pb	CNT	–COOH	50	9003
BNNT@50Pb	BNNT	–	50	9016
BNNTCOO^-^@50Pb	BNNT	–COO^-^	50	9005
BNNTCOOH@50Pb	BNNT	–COOH	50	8990

To have a detailed insight and visualize the output of simulation systems, VMD software is used^31^.

## Result and discussion

To understand the adsorption process of lead ions on the surface of two nanotubes (CNT and BNNT) and the effect of –COO– and –COOH functional groups on the adsorption behavior of these ions, twelve systems have been designed and investigated.

**Figure 3:**
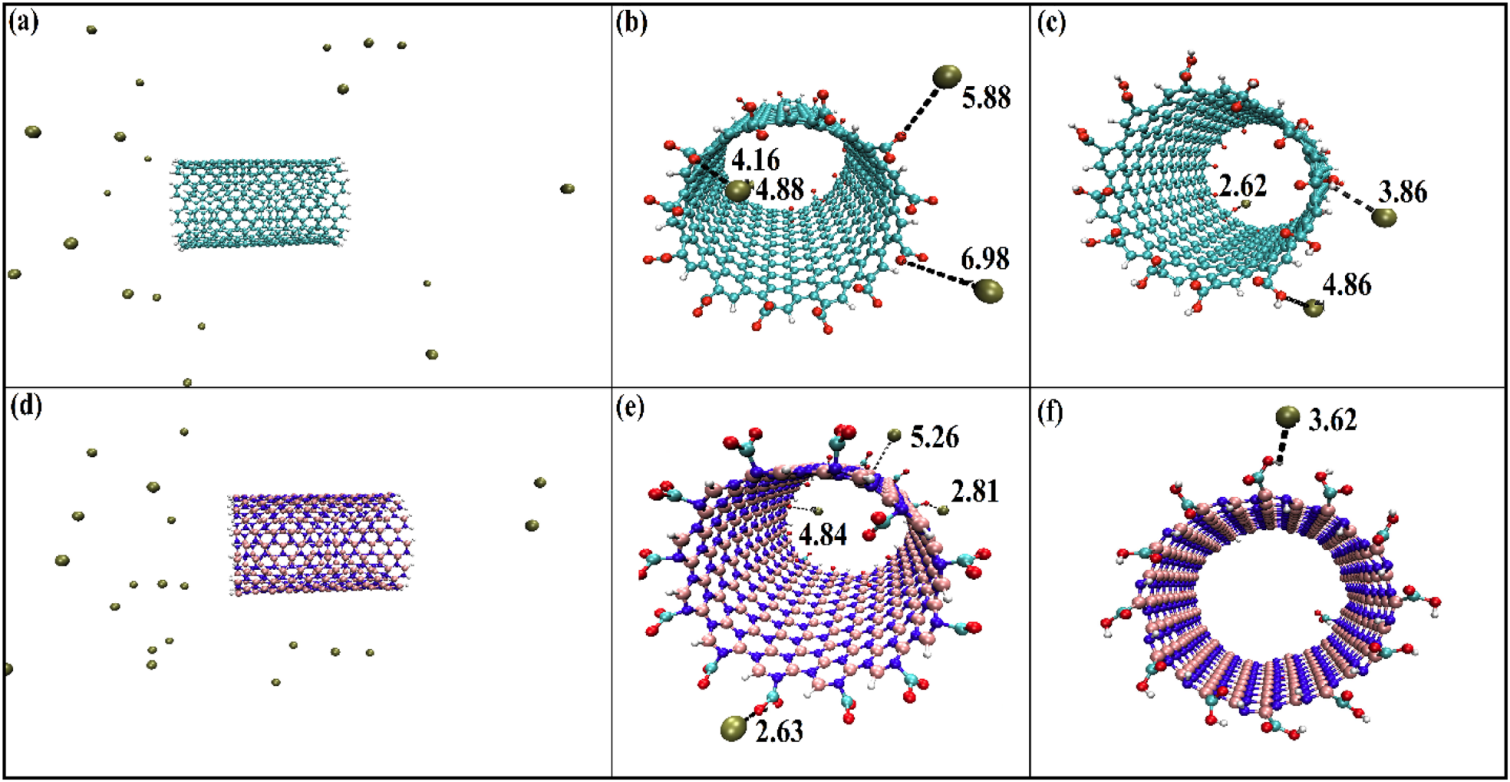
Snapshots of Pb^2+^ ions with nanotubes: (**a**) CNT, (**b**) CNTCOO^−^, (**c**) CNTCOOH, (**d**) BNNT, (**e**) BNNTCOO^−^, (**f**) BNNTCOOH (systems with 20 Pb ions).

The adsorption of ions on the mentioned nanotubes has been carefully studied using the MD simulation, and the final snapshots of several systems with 20 Pb ions after reached equilibrium is shown in Fig. 3.

As is obvious from this Figure, no lead ions are adsorbed on the pristine nanotubes, whereas, after functionalization of the CNT nanotube with –COO– and –COOH, 4 and 3 lead ions have been adsorbed, respectively (panel A). Furthermore, in the case of the BNNTCOO- and BNNTCOOH, adsorbed lead ions are four and one, respectively. Our results show that, the –COO– functional group has significantly improved the performance of nanotubes in the adsorption process, which can be due to the negative charge of the –COO– group, that adsorbs positively charged lead ions. Also, by comparing the number of adsorbed ions on CNTCOOH and BNNTCOOH, it can be concluded that CNT exhibits better adsorption behavior than BNNT at low pH.

### Energy

#### Investigation of energy in systems with 20 Pb ions

**Figure 4:**
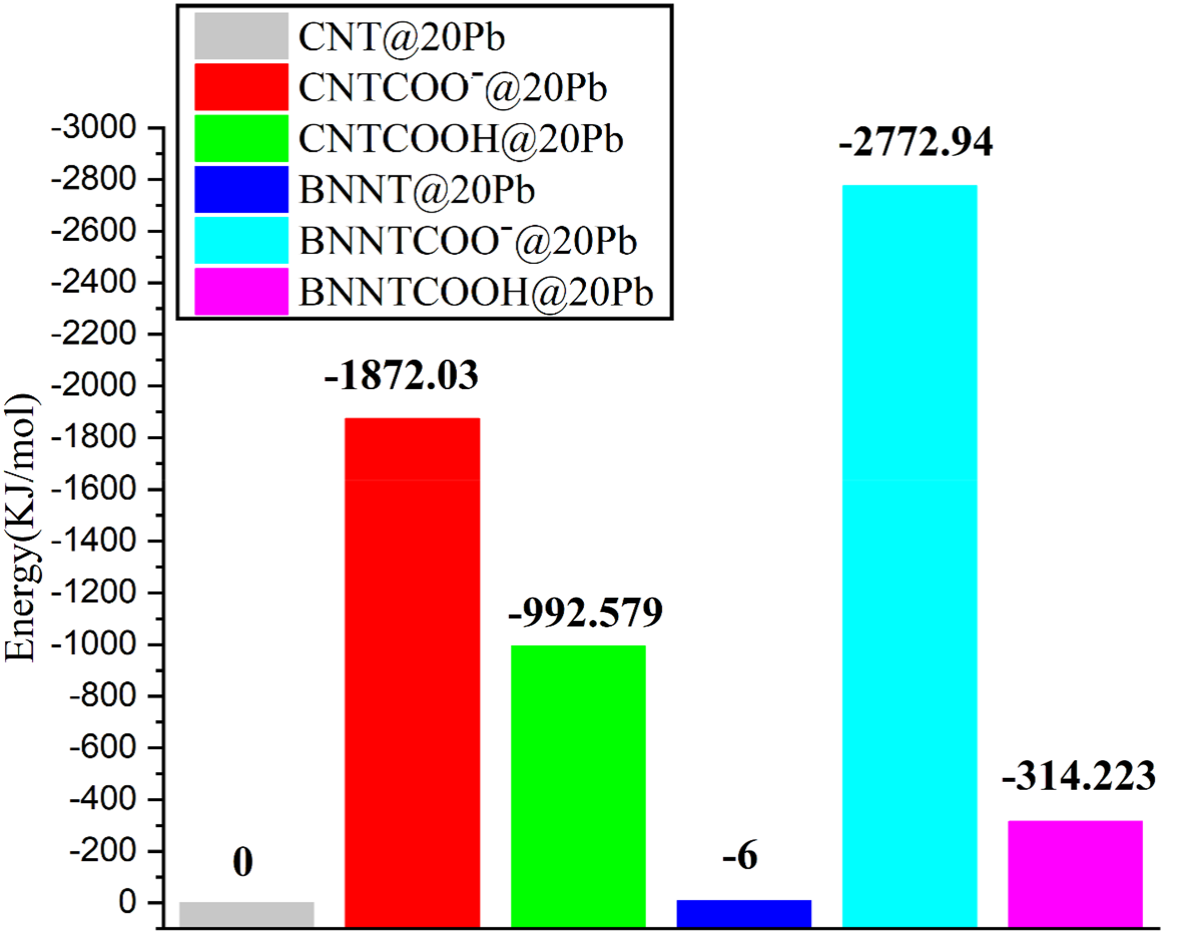
Average electrostatic energy between the nanotubes and the ions of the systems with 20 Pb ions.

To provide valuable insights into the ion’s adsorption behavior at the microscopic level, the electrostatic energy of the simulation systems is calculated. Interaction energies are extracted from MD productions to explain the adsorption process. Interaction energies between nanotubes and ions in systems with 20 Pb ions are given in Fig. 4.

As can be seen from this figure, all the calculated energies are negative, or in other words, the adsorption process is exothermic. Our results show that the stability order of the studied systems for CNT is CNTCOO– > CNTCOOH > CNT, and for BNT systems follows the order BNNTCOO– > BNNTCOOH > BNNT. As can be seen, the least electrostatic energy value is obtained for pristine nanotubes, and it becomes more negative upon functionalization of the nanotubes. The negatively charged functionalized nanotubes’ surface favors electrostatically adsorbing the Pb ions more than the pristine nanotubes without surface charge. It is observed that the interaction of Pb ions with the functionalized nanotubes is stronger than pristine nanotubes and the ions exhibits the most tendency for the adsorption on the CNTCOO– in terms of the electrostatic energy values. This fact can be attributed to the existence of the negative charge of the –COO– functional group, which causes more adsorption of positively charged lead ions on the CNTCOO– nanotube. The comparison between the electrostatic energies of BNNTCOO– and CNTCOO– shows that the interaction energy for BNNTCOO– is about 900 kJ/mol more negative than CNTCOO– (see Fig. 4), which confirms the better adsorption behavior of BNNTCOO– in removing Pb ions from polluted water. The obtained results show that the adsorption energies are significantly dependent on the functional group, such that the COO-causes both nanotubes perform better in adsorbing ions in comparison to pristine ones.

#### Investigation of energy in systems with 50 Pb ions

**Figure 5:**
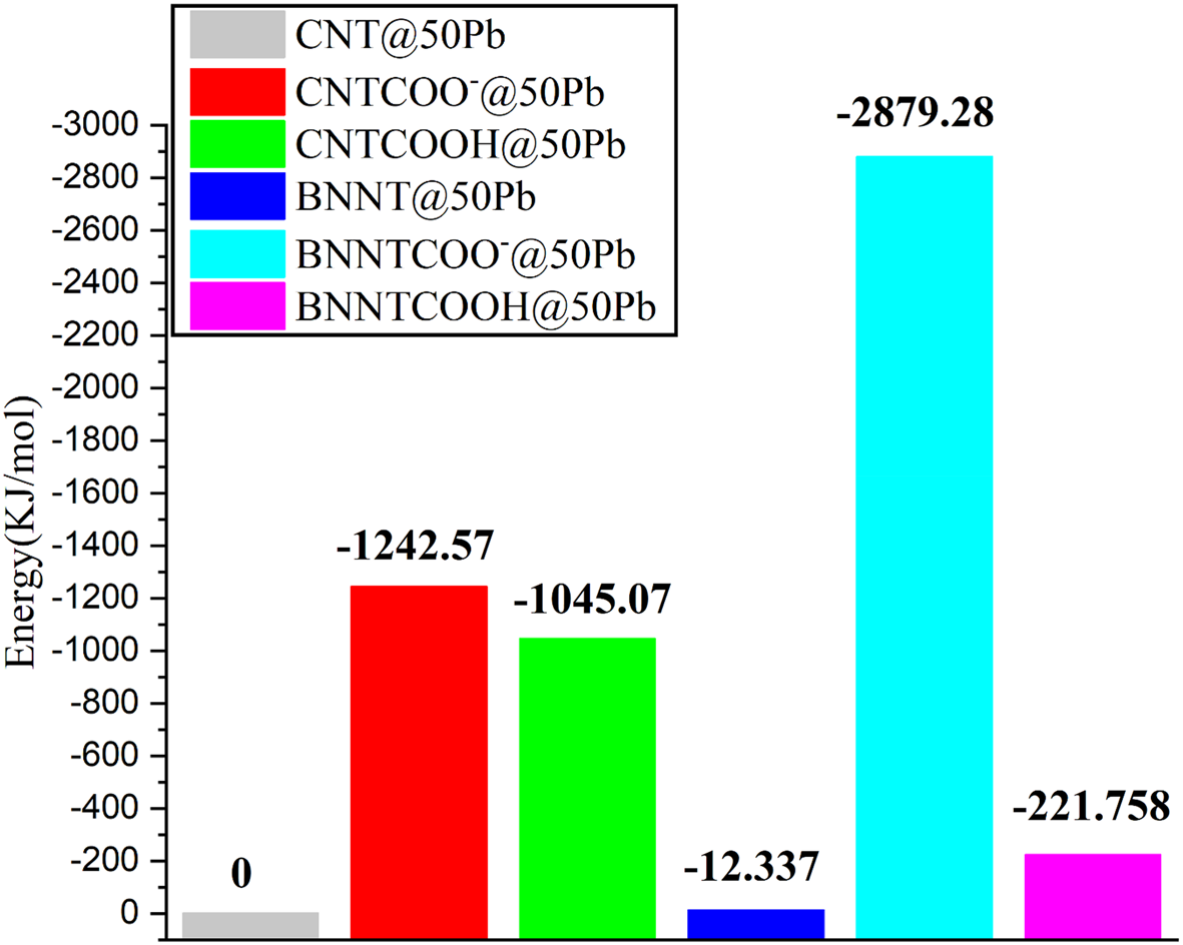
Average electrostatic energy between the nanotubes and the ions of the systems with 50 Pb ions.

To investigate the performance of nanotubes in the process of adsorbing ions at different concentrations, the electrostatic energy of systems with 50 Pb is calculated and results are shown in Fig. 5.

Presented results in Fig. 5 show that the stability of the studied CNT nanotube systems follows the order CNTCOO– > CNTCOOH > CNT, and the same trend can be observed for BNNT nanotubes, BNNTCOO– > BNNTCOOH > BNNT. These results confirm that the –COO– functionalized nanotubes have the most negative energy value, which is consistent with the obtained results from the energy values of the systems with 20 Pb ions.

According to the obtained results, in systems with a more concentration (50 Pb ions), as in the previous system, the interaction between the ions and CNTCOOH is stronger than the ions with BNNTCOOH. Furthermore, as the concentration of ions increases, the interaction between ions and CNTCOOH becomes more negative, which can be related to the increase in the number of adsorbed ions. Surprisingly, the interaction of ions with BNNTCOOH becomes weaker in the system with higher concentration, which can be caused by the weak adsorption of ions on the nanotube and the repulsion between them due to the increased concentration of ions. The functional group with negative charge completely changes the adsorption behavior of nanotubes so that BNNTCOO– shows better performance than CNTCOO– nanotube. Furthermore, it can be noted that with increasing concentration, the BNNTCOO– system becomes more stable by approximately 106 kJ/mol, while the adsorption energy of the CNTCOO– system becomes more positive almost ~ 629 kJ/mol.

### Radial distribution function (RDF)

**Figure 6:**
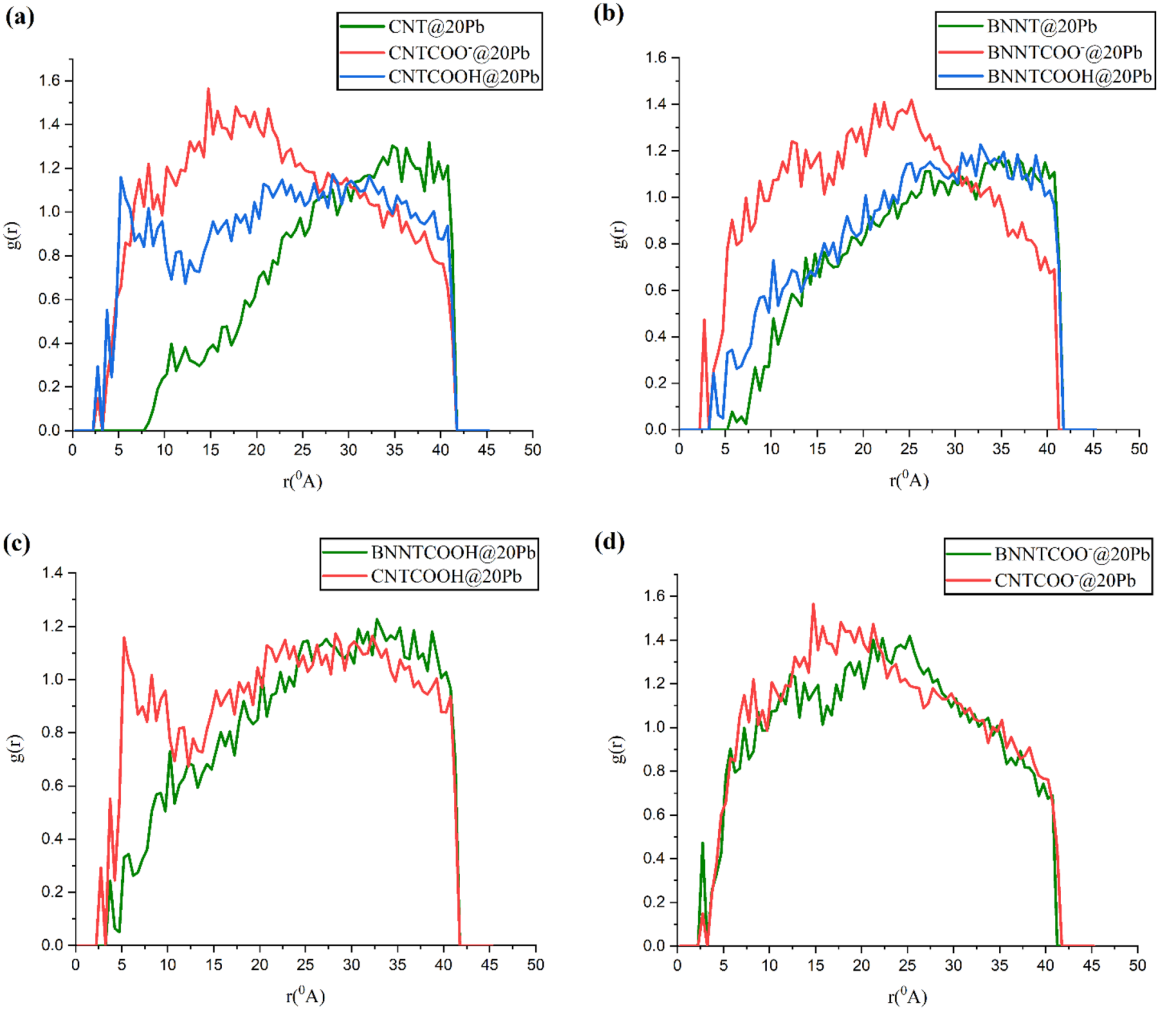
Radial distribution function (RDF) between the ions and the nanotubes of the systems with 20 Pb ions.

The radial distribution function (RDF) indicates the probability of finding ions in a spherical shell with a certain thickness at a distance (r) from nanotubes. RDF analysis is a benefit for understanding the interaction of ions with nanotubes in the studied system and can provide information about the position and distribution of these ions. The RDF in the MD simulation is calculated using the following formula^32^:

1 $$g\left( r \right) = \frac{1}{{\rho_{j} N_{i} }}\sum\nolimits_{i}^{{N_{i} }} {\sum\nolimits_{j}^{{N_{j} }} {\frac{{\delta \left( {r_{ij} - r} \right)}}{{4\pi r^{2} }}} }$$

where ${\rho }_{j}$ is bulk density of particles j and ${N}_{i}$ is number of particles i. The RDF for the systems with 20 Pb ions is calculated and the results represented in Fig. 6.

According to Fig. 6a, the range of the RDF diagram in the pristine CNT system is less than the functionalized ones, and it does not show any interaction with Pb ions up to about 0.8 Å. On the other hand, the strongest peak in the RDF diagram of CNT systems belongs to CNTCOO-, which indicates the interaction of this nanotube with ions is the most. The comparison between the RDF diagrams in Fig. 6c shows that the peak intensity of the RDF graphs for the CNTCOOH system at the distance r = 5 Å is significantly more than that of the BNNTCOOH system. This fact indicates that in acidic conditions, the ions are positioned at a nearer distance from the CNT in comparison to BNNT. The RDF pattern in Fig. 6d shows that the probability of existence ions at a distance of 0.27 nm around BNNTCOO– is more than that of CNTCOO–. In other words, in the presence of COO^−^ functional group, nanotube BNNT shows a better performance in adsorbing ions. All the obtained results from the RDF calculations are consistent with the obtained results from snapshots and electrostatic energy values.

### MSD

The diffusion coefficients of ions ($${D}_{ion})$$ are calculated from mean square displacement (MSD) using Eqs. (2) and (3)^18^:

2 $$MSD\left( t \right) = \frac{1}{N}\sum\nolimits_{i = 1}^{N} {\left( {\left| {r_{i} \left( {t + \Delta t} \right) - r_{i} \left( t \right)} \right|} \right)^{2} }$$

3 $$D_{ion} = \frac{1}{6} \mathop {\lim }\limits_{t \to \infty } \frac{d}{dt}\left( {MSD} \right)$$

where r i (t) is the vector position of the ion i at time t and N is the number of ions.

The MSD is influenced by different factors such as the type of material, the concentration of ions, the presence of functional groups, and the interaction energy^33^.

Since only one ion type is studied in this work, the effect of the material type has not been investigated. Diffusion coefficients for ions in all systems are given in Table 2.

**Table 2:** Average diffusion coefficient (D_i_) of Pb ions in the investigated systems.

System	D_i_ (10^–5^ cm^2^/s)
CNT@20Pb	0.0205 (+ /− 0.0007)
CNTCOO^-^@20Pb	0.0191 (+ /− 0.0032)
CNTCOOH@20Pb	0.0198 (+ /− 0.0022)
BNNT@20Pb	0.0268 (+ /− 0.0039)
BNNTCOO^−^@20Pb	0.0250 (+ /− 0.0050)
BNNTCOOH@20Pb	0.0148 (+ /− 0.0031)
CNT@50Pb	0.0048 (+ /− 0.0008)
CNTCOO^−^@50Pb	0.0080 (+ /− 0.0017)
CNTCOOH@50Pb	0.0081 (+ /− 0.0004)
BNNT@50Pb	0.0083 (+ /− 0.0006)
BNNTCOO^−^@50Pb	0.0069 (+ /− 0.0002)
BNNTCOOH@50Pb	0.0080 (+ /− 0.0013)

**Figure 7:**
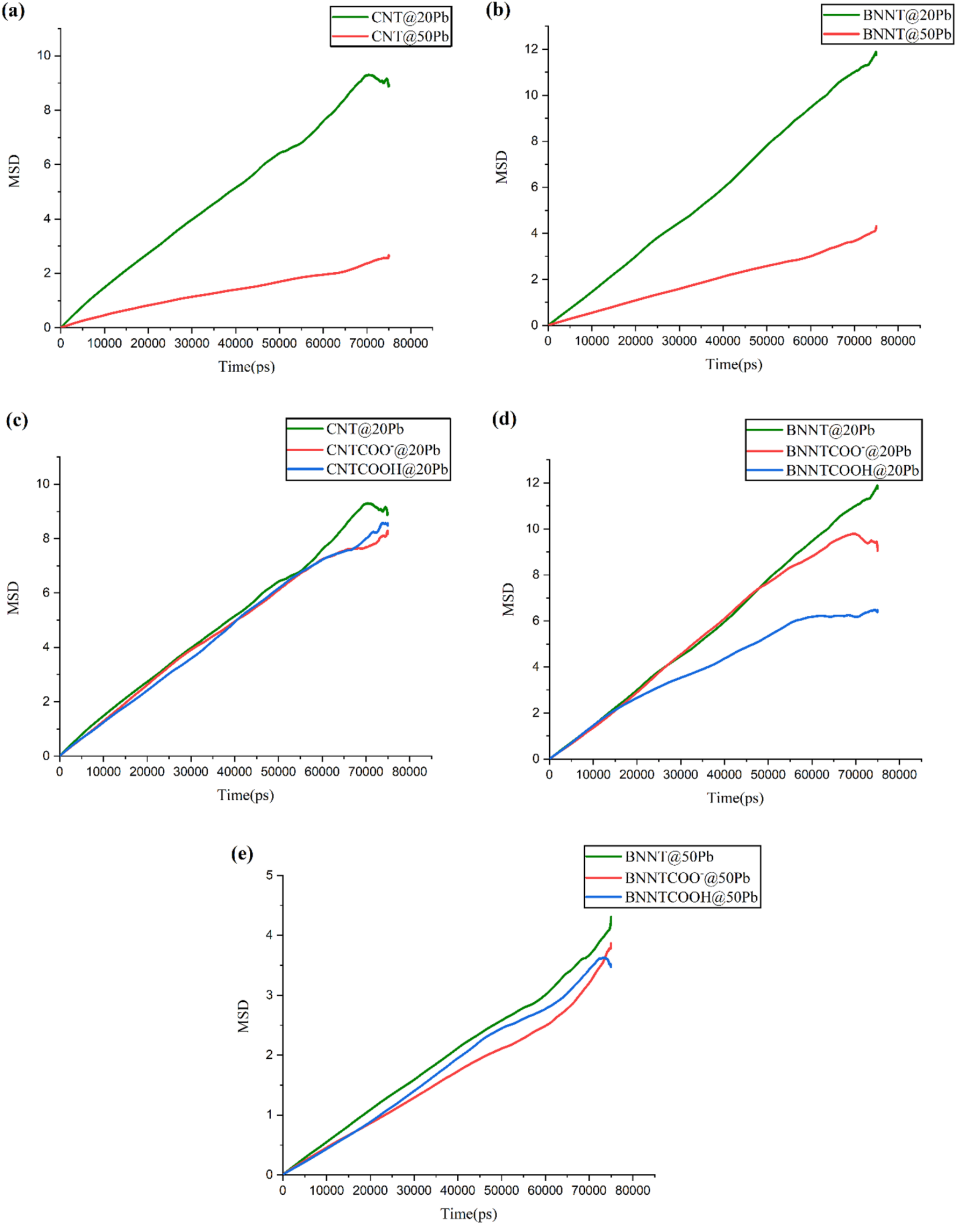
Mean square displacement (MSD) of ions in the investigated systems.

To investigate the effect of concentration, the MSD plots of Pb ions in 20CNT, 50CNT, 20BNNT, and 50BNNT systems as a function of simulation time are plotted in Fig. 7a,b.

Close inspection of Fig. 7a,b reveals that the ions in systems with 20 Pb ions have more displacement than systems with 50 Pb ions, which is related to the better adsorption of ions at high concentrations.

The obtained results in Table 2 show that the values of the diffusion coefficient in systems with 20 Pb ions are more than those with 50 Pb ions, which confirms the adsorption capacity increases in higher concentrations (Similar to the results of Fig. 7a,b). These results are similar to the obtained results from the work’s Anitha et al.^18^ which show that D values decrease with increasing concentration. MSD plots for 20CNT, 20CNTCOO-, 20CNTCOOH, 20BNNT, 20BNNTCOO- and 20BNNTCOOH systems are investigated for elucidation of the effect of functional groups on ion diffusion (Fig. 7c,d).

As seen in Fig. 7c,d, the slope of MSD curves in functionalized nanotubes in comparison to pristine ones is lower, indicating that the functional group is resulting in a local restriction and a decreased diffusion of ions. This fact may be related to the strong interaction between the ions and functionalized nanotubes, which can be caused slower movement of ions.

The effect of interaction energy on the mobility of Pb ions can be elucidated by investigating the MSD plots of 50BNNT, 50BNNTCOO–, and 50BNNTCOOH systems (see Fig. 7e). The diffusivity of ions in the BNNTCOO– system is much reduced as compared with BNNT and BNNTCOOH systems, which can be attributed to their more electrostatic interaction with the adsorbent (see Fig. 7e). Figure 7e shows that the MSD curve of the BNNT system has a steeper slope than the BNNTCOOH system, which is directly related to the lower interaction energies between ions and BNNT.

The interaction energy in the 50BNNTCOO– system is maximum, so the diffusion coefficient of ions in this system becomes minimum in comparison to the other BNNT systems. (See Table 2). Furthermore, the stronger interaction between Pb ions and nanotube in the 50BNNTCOOH system in comparison to pristine BNNT caused a decrease in its diffusion coefficient. These results correspond to the outcomes of Chopra et al.^34^, which have shown the diffusion coefficient decreases with increasing interaction energy.

## Conclusion

In this study, molecular dynamics simulation is used to investigate the adsorption behavior of Pb ions on carbon and boron nitride nanotubes. Also, the effect of –COO– and –COOH functional groups on the adsorption process is evaluated. The nanotube’s adsorption performance is studied on two different concentrations of Pb ions (20 and 50 lead ions). Various analyzes, such as interaction energy, MSD, and RDF, have been examined. Inspection of the MSD diagrams and diffusion coefficients showed that the MSD curve is influenced by various factors, suchlike ion concentration, functional group, and interaction energy. The obtained results confirmed that the functionalization of nanotubes increases the electrostatic energy and decreases the diffusion of ions. Comparing the energy of functionalized systems reveals that upon functionalization of nanotubes with the –COO–, the electrostatic energy becomes more negative compared to the –COOH functional group. The negative charge of the –COO– functional group can be caused a stronger attraction of ions, increase the adsorption capacity of the nanotube, and decrease the ions’ diffusion coefficient. Furthermore, the diffusion coefficients of ions in the studied systems decrease with increasing their concentration. Our obtained results proved that the functionalization of carbon and boron nitride nanotubes with –COO– increases their performance for adsorbing Pb ions. Hence, they can be suitable for removing heavy metals from contaminated waters.

## Data Availability

The datasets used and/or analysed during the current study available from the corresponding author on reasonable request.
